# Supplementary figures and images for: Correlative Study on Impaired Prostaglandin E2 Regulation in Epicardial Adipose Tissue and Its Role in Maladaptive Cardiac Remodeling via EPAC2 and ST2 Signaling in Overweight Cardiovascular Disease Subjects
Source: Int J Mol Sci. 2020 Jan 14;21(2):520. doi: 10.3390/ijms21020520 (PMC7014202; doi:10.3390/ijms21020520)

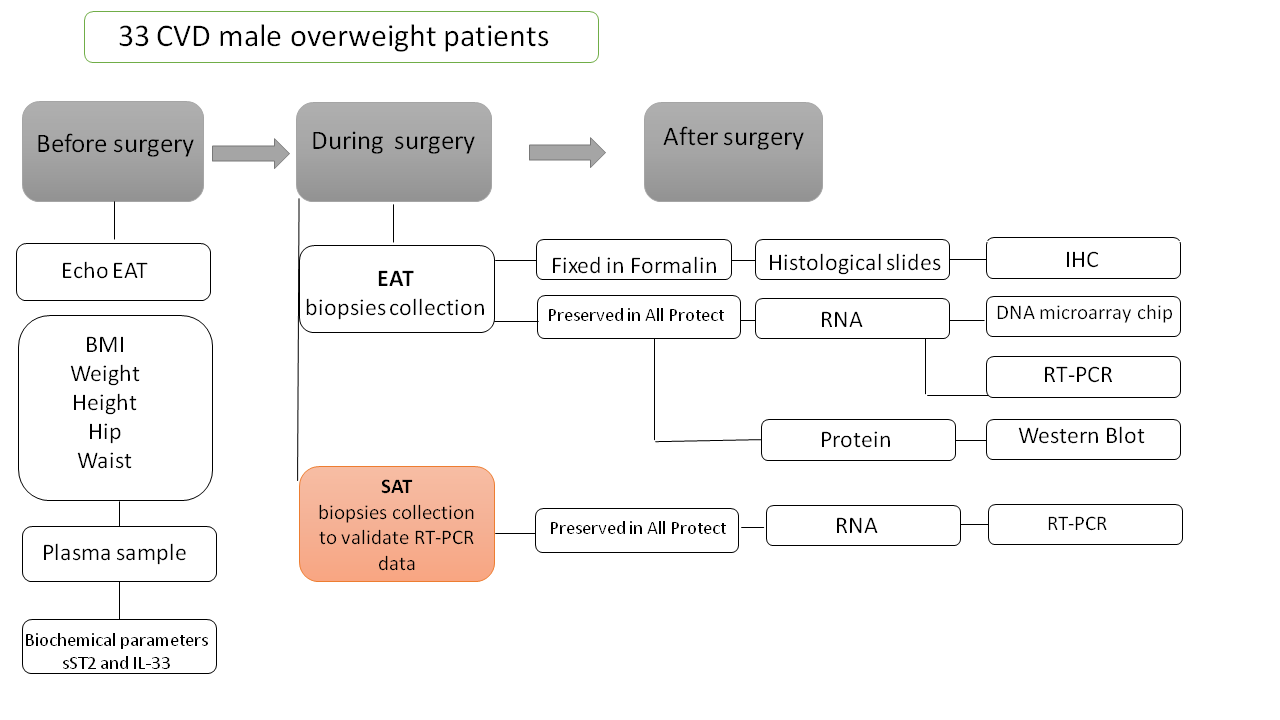

Supplement: Supplementary file 1 [file ijms-21-00520-s001.zip › ijms-689757 suppl for proof/Supplementary Figure S1.tif]

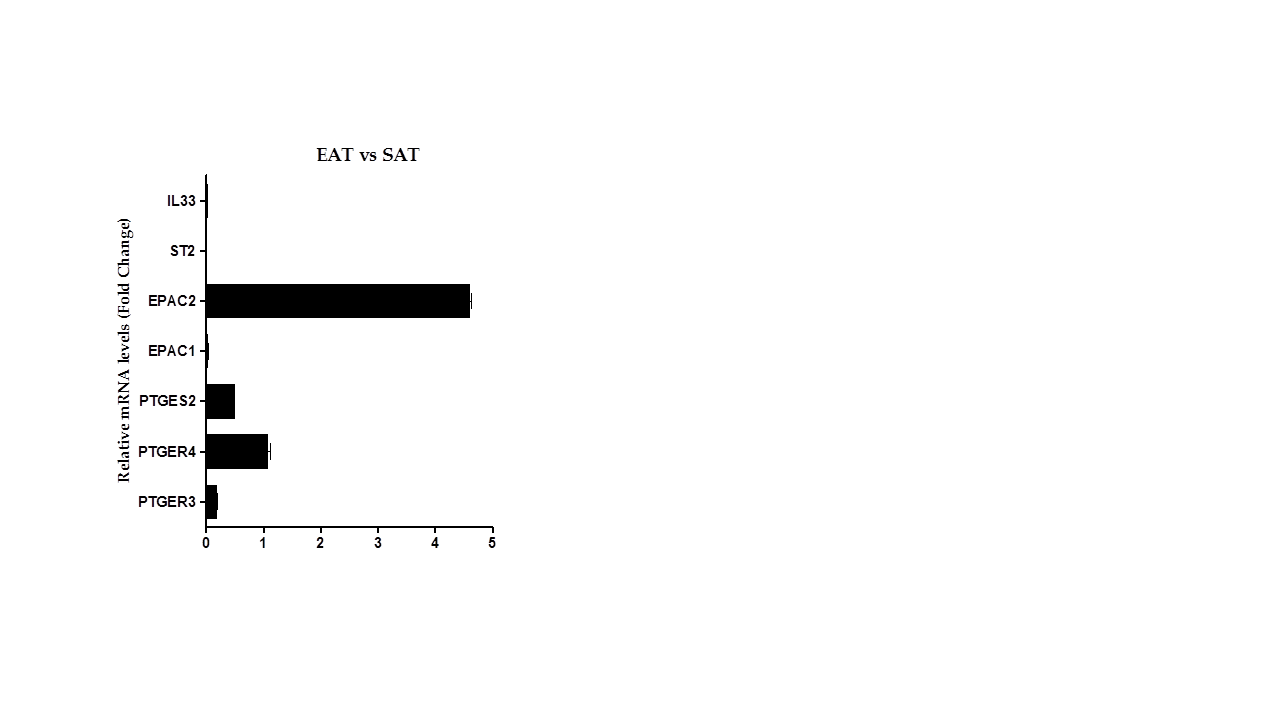

Supplement: Supplementary file 1 [file ijms-21-00520-s001.zip › ijms-689757 suppl for proof/Supplementary Figure S2.tif]

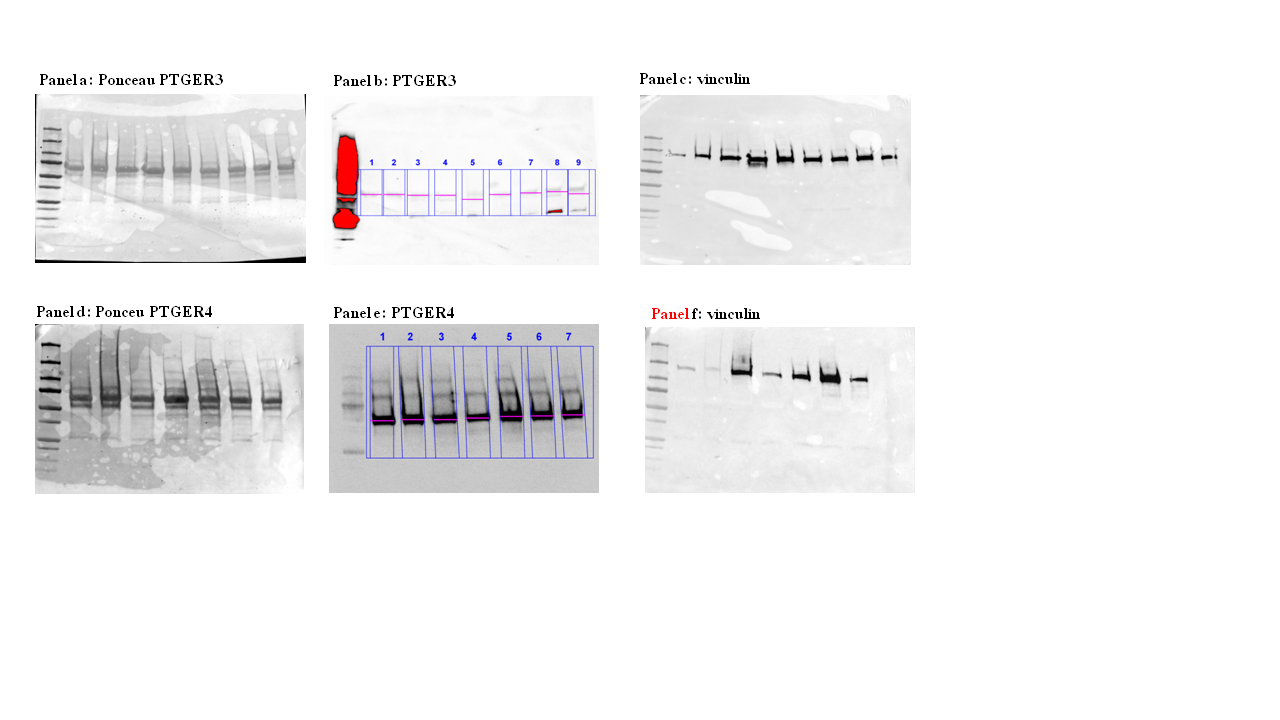

Supplement: Supplementary file 1 [file ijms-21-00520-s001.zip › ijms-689757 suppl for proof/Supplementary Figure S3.tif]
